# Supplementary material for: Nonlinear mechanics of hybrid polymer networks that mimic the complex mechanical environment of cells
Source: Nat Commun. 2017 May 25;8:15478. doi: 10.1038/ncomms15478 (PMC5458517; doi:10.1038/ncomms15478)
Supplement: Supplementary Information — Supplementary Figures, Supplementary Methods and Supplementary Reference [file ncomms15478-s1.pdf]

Supplementary Methods

Carbon Nanotube characterisation. The size of the carbon nanotubes was characterized by transmission electron microscopy (TEM). Measurements were performed with a JEOL JEM 1010 microscope with an acceleration voltage of 60 kV and a charged coupled device (CCD) camera. The samples were prepared by placing 5 µL of a 0.05 mg mL^{-1} carbon nanotube dispersion in water on a carbon-coated copper grid (200 mesh, EM science). The grid was air-dried for at least 24 hours at room temperature before measuring. Analysis of the images to obtain the nanotube diameter was performed using ImageJ software. We find an average diameter of 1.8 nm for the SWCNTs and a much larger average diameter of 20 nm for the MWCNTs (Supplementary Figure 2). The length of both nanotubes is in the order of 10 micrometres. We also studied the effect of shorter SWCNTs, which have a length in the order of 1 µm and a similar diameter to the longer SWCNTs (Supplementary Figure 2). In the TEM images we do not observe aggregation of the nanotubes, indicating that they are well dispersed in solution.

Synthesis of AKQAGDV-functionalized polyisocyanides. The synthetic scheme is given in Supplementary Figure 8; numbered compounds refer to this Figure. BCN-amine compound **1** (Synaffix, 57 mg, 0.17 mmol) and *N*-succinimidyl-4-maleimidobutyrate (45 mg, 0.16 mmol) were dissolved in DMSO (1 mL). K_2CO_3 (22 mg, 0.16 mmol) was added and the mixture was stirred for one hour at room temperature. The formation of BCN-maleimide compound **2** was confirmed by mass spectrometry. MS cal.: 489.6, obtained: 490.2.

The CAKQAGDV peptide (JPT Peptide Technologies, 25 mg, 31.6 µmol) was dissolved in phosphate buffer (pH 7.2, 1 mL). This peptide solution (63.3 µL, 2.0 µmol), the BCN-maleimide reaction mixture (12.2 µL, 2.0 µmol) and acetonitrile (121 µL) were mixed and stirred for 24 h at 5 °C. The formation of the BCN-peptide compound **3** was checked by TLC and mass spectrometry and the compound was used without further workup. MS cal.: 1280.5, obtained: 1280.4.

Azide-functionalized PIC (0.18% azide functionalization, determined by a dye test[1]) was dissolved in acetonitrile (2.5 mg mL^{-1}) and 2.0 equivalents of the BCN-functionalized AKQAGDV peptide were added. The mixture was stirred for 24 h at T = 5 °C. The polymers were precipitated in diisopropyl ether, filtered and air-dried to obtain the peptide-functionalized PICs in a yield of 87%.

Synthesis of dibenzocyclooctyne-functionalized acrylamide. The synthetic scheme is given in Supplementary Figure 13; numbered compounds refer to this Figure. Dibenzocyclooctyne-amine (DBCO-amine) compound **4** (Click Chemistry Tools, 12 mg, 0.044 mmol) was dissolved in dry THF (10 mL). Acryloyl chloride (13.4 µL, 0.13 mmol) and a drop of triethylamine were added and the reaction mixture was stirred for two hours at room temperature after which the mixture was poured into ice water (50 mL). The water mixture was extracted with dichloromethane (3x 50 mL), the combined organic layers were washed with a 0.1 M HCl solution, dried with $MgSO_4$ and the solvent was evaporated *in vacuo*. The resulting product was purified by repeated precipitation in *n*-hexane and vacuum dried to yield 4.4 mg (30%) of compound **5** as a yellow solid.

^{1}H-NMR (400 MHz, $CDCl_3$, ppm) δ 7.67 (d, 1H, CH_{Ar}), 7.38 (m, 7H, CH_{Ar}), 6.28 (s, 1H, NH), 6.10 (m, 1H, CO-C$\underline{H}$-CH_2), 5.92 (m, 1H, CO-CH-C$\underline{H}_2$), 5.53 (d, 1H, CO-CH-C$\underline{H}_2$), 5.16 (d, 1H, CH_{Ar}-C$\underline{H}_2$), 3.72 (m, 1H, CH_{Ar}-C$\underline{H}_2$), 3.46 (m, 1H, CO-CH_2-C$\underline{H}_2$), 3.29 (m, 1H, CO-CH_2-C$\underline{H}_2$), 2.56 (m, 1H, CO-C$\underline{H}_2$-CH_2), 2.00 (m, 1H, CO-C$\underline{H}_2$-CH_2) ^{13}C NMR (101 MHz, $CDCl_3$, ppm) δ 170.09, 164.43, 151.33, 148.35, 132.38, 131.58, 129.53, 128.95, 128.25, 128.07, 127.73, 126.83, 124.92, 122.46, 121.44, 114.29, 108.02, 54.86, 48.60,

35.04, 34.12. FT-IR (ATR, cm^{-1}) 3308, 2925, 1658, 1448, 1400, 1230, 754. MS cal.: 330.4, obtained: 331.1.

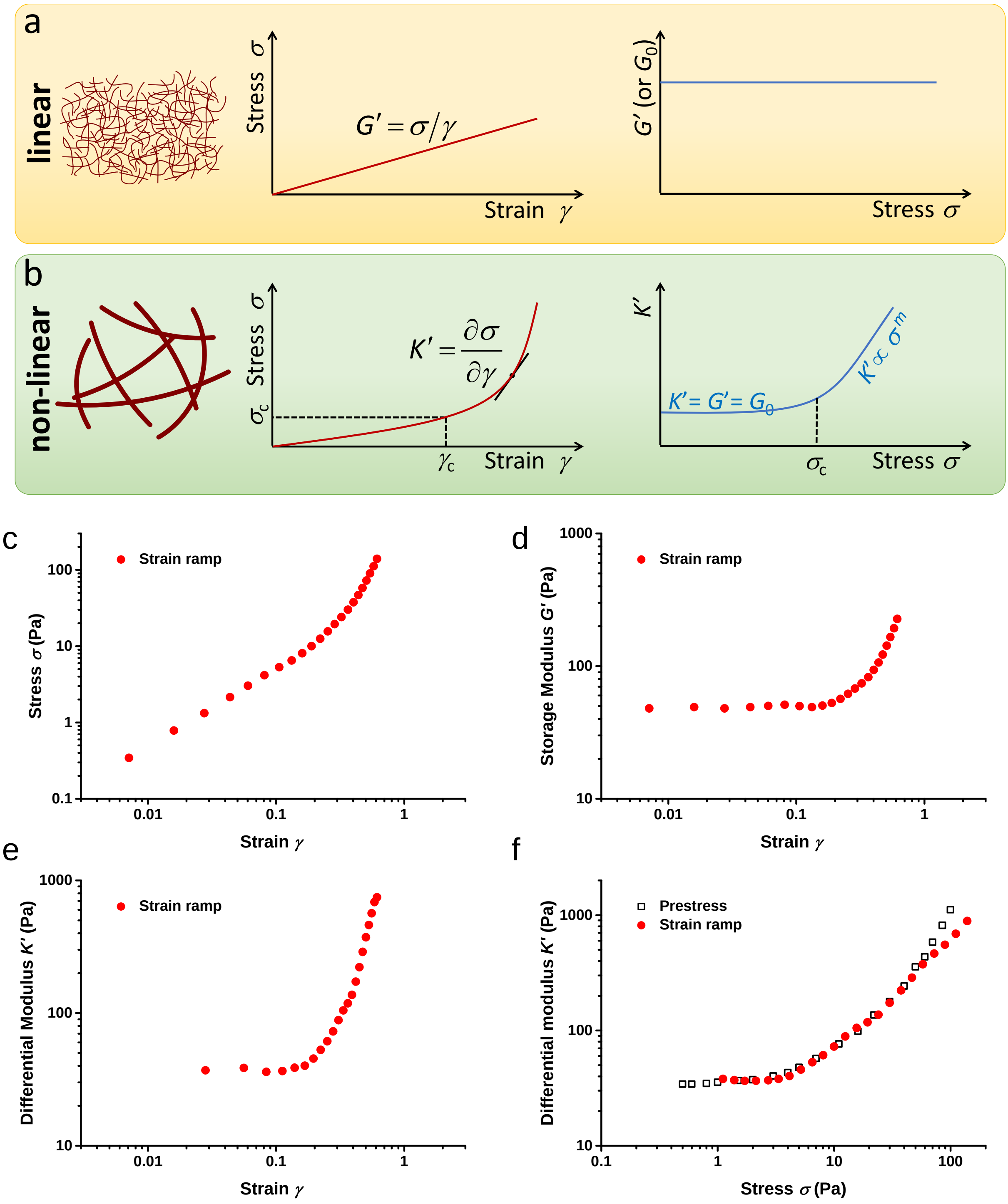

Supplementary Figure 1 | Schematic overview of the mechanical parameters in materials with a linear (a) and a nonlinear strain-stiffening (b) response. (c-f) Nonlinear mechanical properties of a PIC hydrogel (no composite) measured with a classical strain sweep (red circles) and using the pre-stress protocol (black open squares) in different representations of σ, G' and K' vs γ or σ.

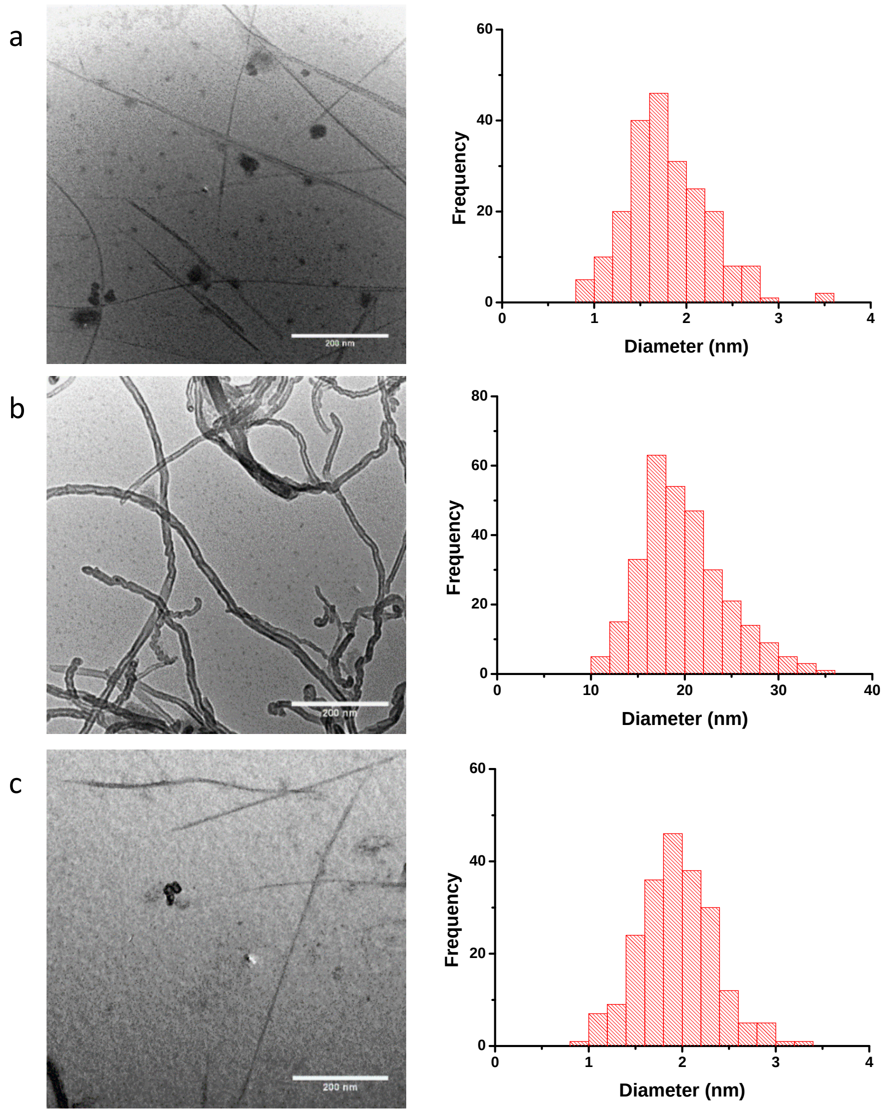

Supplementary Figure 2 | TEM images of SWCNTs (a), MWCNTs (b) and short SWCNTs (c), the scale bar is 200 nm in all images. The average diameter of the nanotubes obtained from these images is 1.8 nm for SWCNT's and 20 nm for MWCNT's.

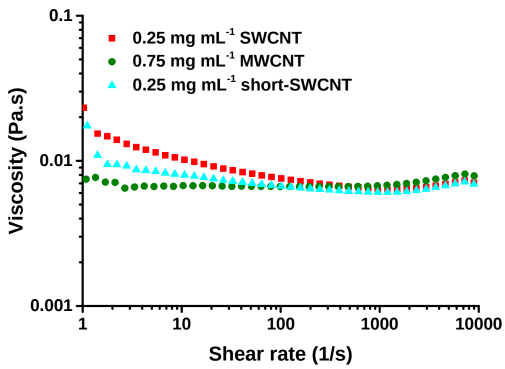

Supplementary Figure 3 | Mechanical properties (viscosity) of CNT solutions as a function of strain rate. At strain rates higher than 10,000 s^{-1}, physical contact between the plates and the sample is lost.

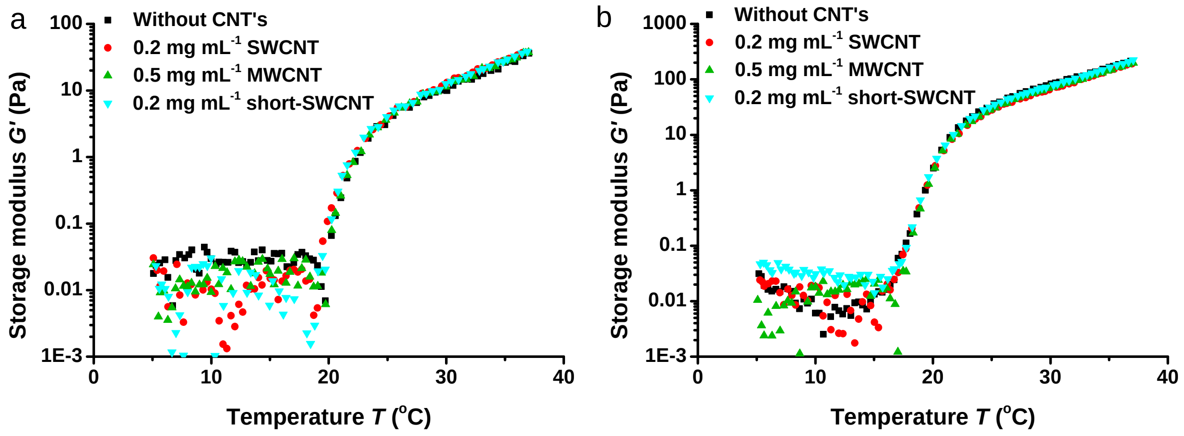

Supplementary Figure 4 | Gelation of the PIC solution upon heating is not affected by the addition of single- or multi-walled carbon nanotubes, as shown by an increase of the storage modulus *G*′ with temperature for (a) 1.0 mg mL^{-1} PIC and (b) 2.5 mg mL^{-1} PIC. NB the (linear) stiffness of the gels at c = 2.5 mg mL^{-1} (b) is higher than the low concentration sample (a).

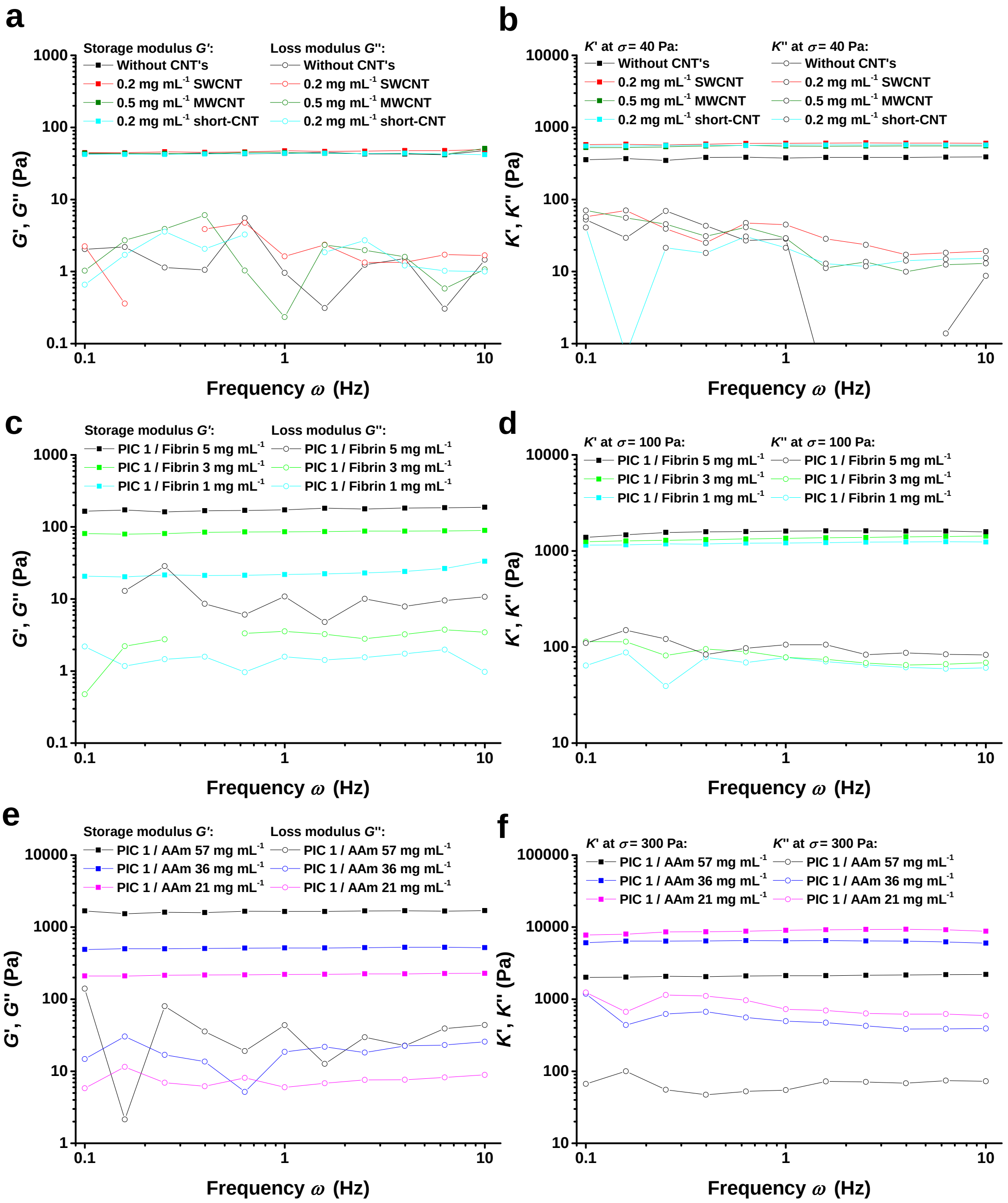

Supplementary Figure 5 | Frequency sweeps of selected materials at low stress (a,c,e) and high stress (b,d,f). The elastic component of the modulus G’ and K’ are constant in the frequency regime that is probed by default. The viscous part is noisy, but also independent of the frequency.

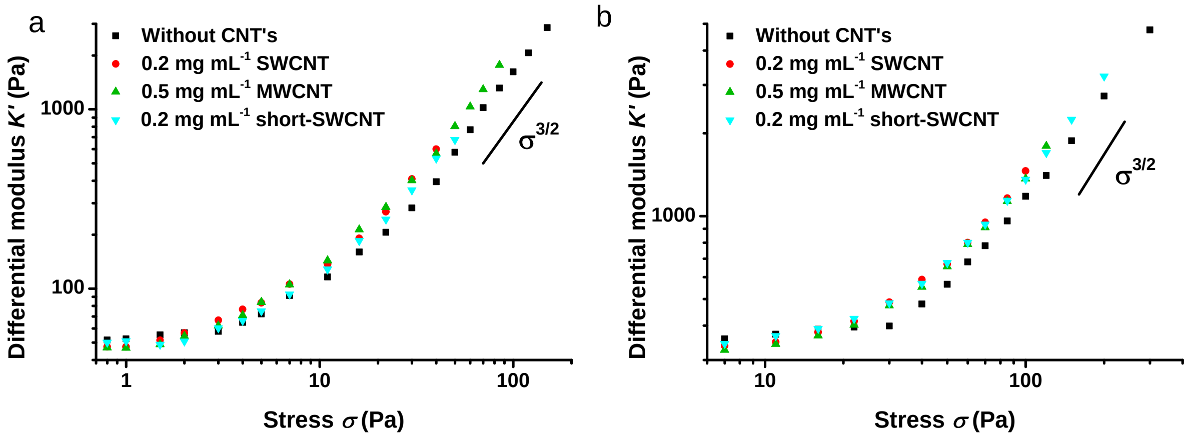

Supplementary Figure 6 | Differential modulus K' as a function of stress σ, showing the stiffening response of PIC hydrogels with and without carbon nanotubes added, with a PIC concentration of (a) 1.0 mg mL^{-1} and (b) 2.5 mg mL^{-1}. K' is independent of σ at low stress and increases with σ at high stress.

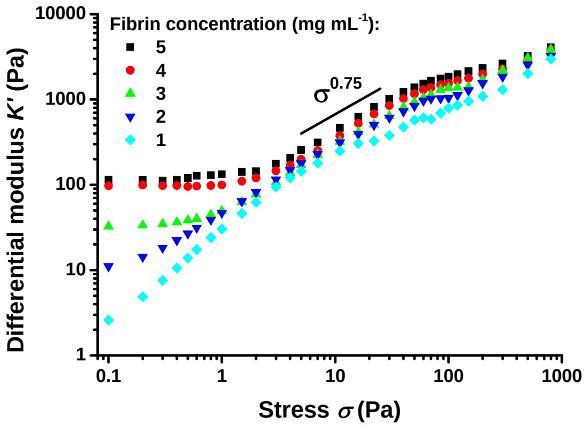

Supplementary Figure 7 | Differential modulus K' as a function of stress σ for fibrin gels with increasing fibrin concentrations. All gels show a similar stiffening response with approximately $K' \sim \sigma^{0.75}$.

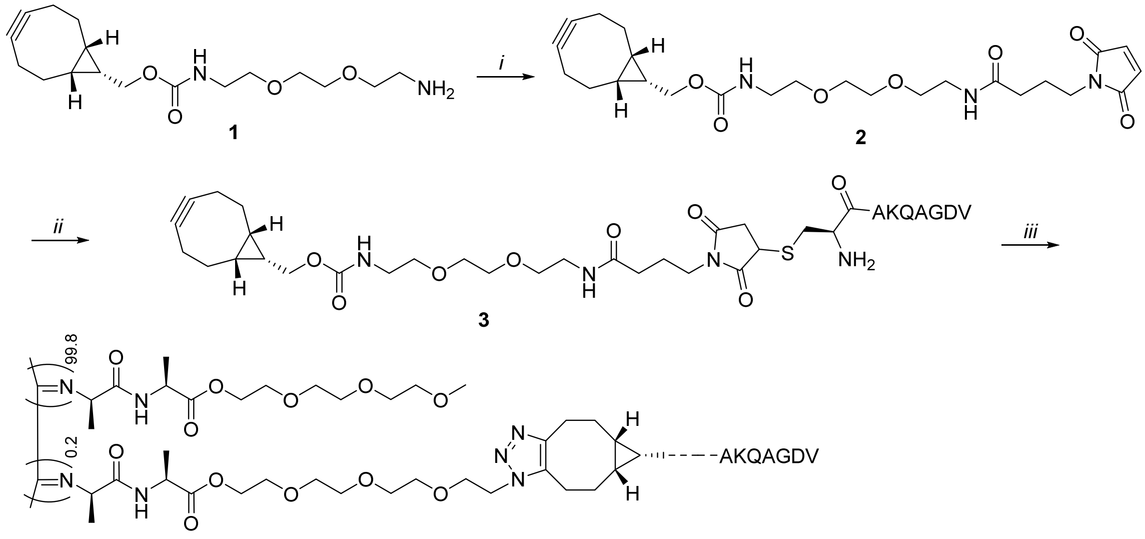

Supplementary Figure 8 | Synthesis of AKQAGDV peptide-functionalized polyisocyanides. *i*) *N*-succinimidyl-4-maleimidobutyrate, K_2CO_3, DMSO, 1 h. *ii*) CAKQAGDV peptide in phosphate buffer, acetonitrile, 24 h. *iii*) $PIC(N_3)_{0.2\%}$, acetonitrile, 24 h, 5 °C.

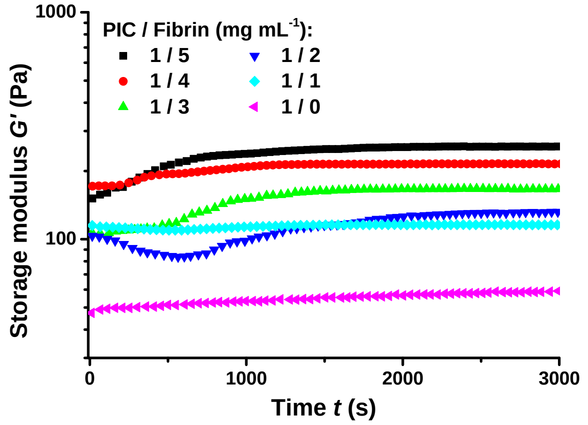

Supplementary Figure 9 | Storage modulus *G*′ as a function of time, showing the formation of the fibrin network in the presence of the peptide-conjugated PIC network at different fibrin concentrations.

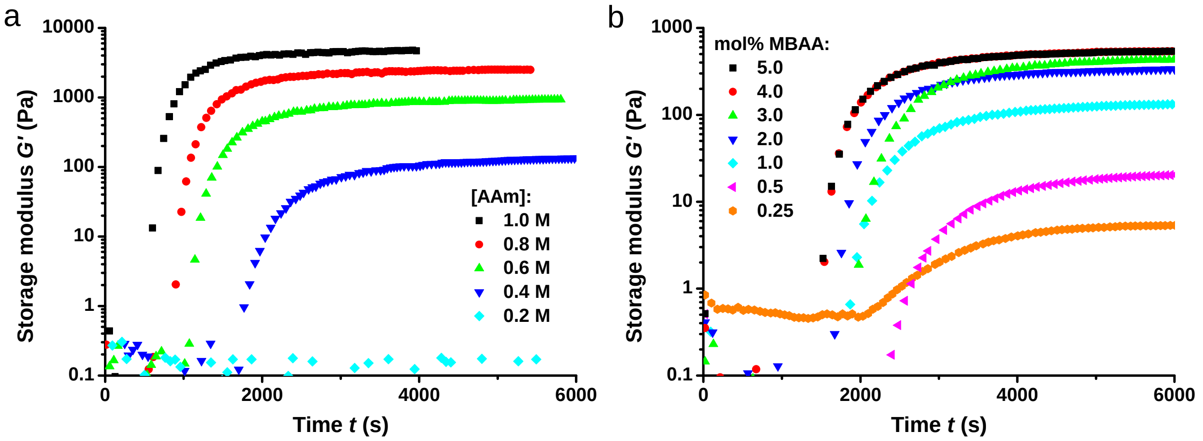

Supplementary Figure 10 | Storage modulus *G*′ as a function of time for (a) PAAm gels with increasing acrylamide concentrations and (b) PAAm gels with increasing cross-linker concentrations at *T* = 50 °C. The modulus increases with increasing acrylamide or cross-linker concentration, for the sample with 0.2 M acrylamide no gelation is observed.

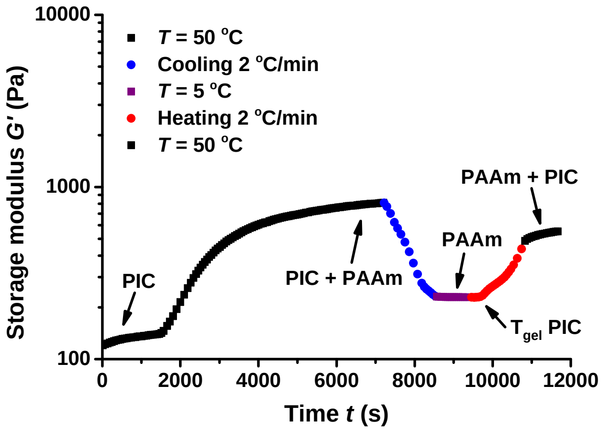

Supplementary Figure 11 | Storage modulus *G′* as a function of time for a PIC/PAAm hybrid hydrogel with 43 mg mL^{-1} AAm (0.6 M) and 1 mg mL^{-1} PIC at different temperatures. The hybrid gel is formed at *T* = 50 °C by polymerization of AAm within the PIC network. Cooling to 5 °C leads to solvation of the PICs but leaves the PAAm network intact. Upon reheating the sample, the PIC network is formed within the PAAm network.

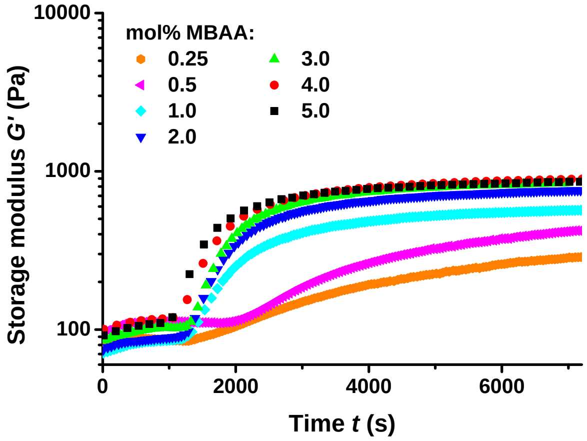

Supplementary Figure 12 | Storage modulus *G′* as a function of time for PIC/PAAm hybrid hydrogels at *T* = 50 °C with 36 mg mL^{-1} (0.5 M) AAm and increasing concentrations of cross-linker MBAA. The final modulus of the gels increases for low cross-linker concentrations, but does not change above 2.0 mol% MBAA.

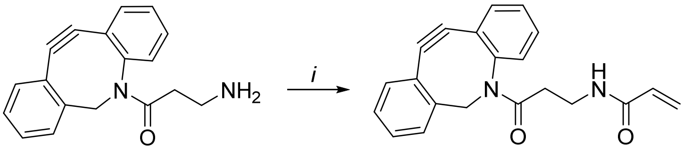

4 5

Supplementary Figure 13 | Synthesis of dibenzocyclooctyne-functionalized acrylamide. *i*) acryloyl chloride, triethylamine, THF, 2 h.

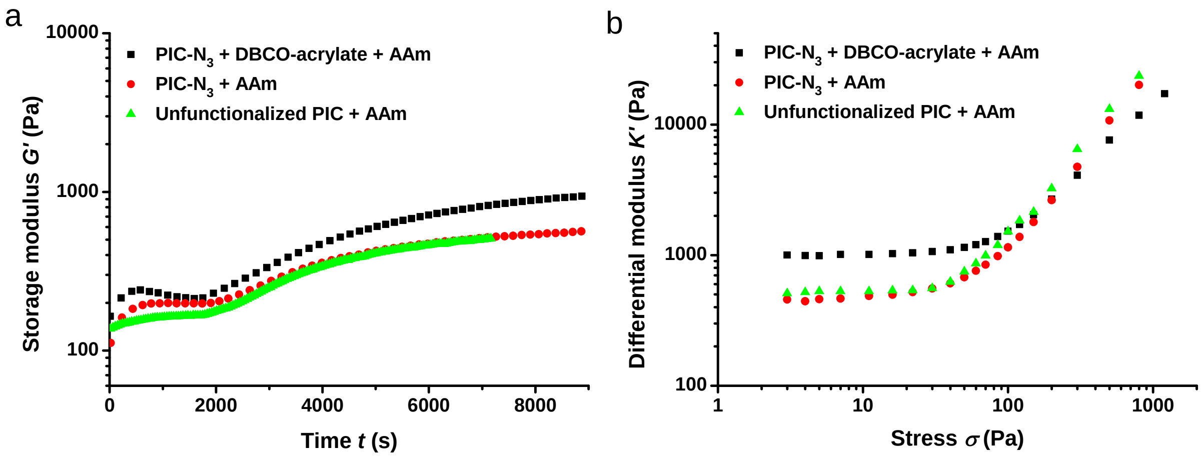

Supplementary Figure 14 | (a) Storage modulus *G′* as a function of time for PIC/PAAm hybrid hydrogels for acrylamide-functionalized PICs (black), azide-functionalized PICs without acrylamide moieties (red) and unfunctionalized PICs (green). The gels with acrylamide-functionalized PICs have a higher stiffness than the other two gels. The PIC and acrylamide concentrations are 1 mg mL^{-1} and 36 mg mL^{-1} respectively for all samples. (b) Differential modulus *K′* as a function of stress for the same PIC/PAAm hybrid gels, also showing a higher linear modulus for the acrylamide-functionalized PICs.

Supplementary reference

1 Das, R. K., Gocheva, V., Hammink, R., Zouani, O. F. & Rowan, A. E. Stress-stiffening-mediated stem-cell commitment switch in soft responsive hydrogels. *Nat. Mater.* **15**, 318-327 (2016).
